# Systematic review of end stage renal disease in Pakistan: Identifying implementation research outcomes

**DOI:** 10.1371/journal.pone.0296243

**Published:** 2023-12-27

**Authors:** Hamad AlRashed, Johanna Miele, Joshua Prasad, Deborah Adenikinju, Chukwuemeka Iloegbu, John Patena, Dorice Vieira, Joyce Gyamfi, Emmanuel Peprah

**Affiliations:** 1 Doctor of Public Health (DrPH) Program, New York University School of Global Public Health, New York, New York, United States of America; 2 Department of Social and Behavioral Sciences, Global Health Program, Implementing Sustainable Evidence-Based Interventions through Engagement (ISEE) Lab, New York University School of Global Public Health, New York, New York, United States of America; 3 New York University Health Sciences Library, New York, New York, United States of America; Delta State University, NIGERIA

## Abstract

**Aim and objectives:**

The aim of this study was to conduct a systematic review analysis to identify and evaluate the available literature on implementation science outcomes research in relation to End Stage Renal Disease (ESRD) in Pakistan.

**Methods:**

A systematic database search of PubMed, Web of Science, EMBASE, Cochrane Library, CINAHL, and Ovid was conducted through October 22^nd^, 2022, without any restrictions on publication dates. A screening and data extraction tool, Covidence, was used to evaluate the literature against our inclusion and exclusion criteria. Furthermore, a Mixed Methods Appraisal Tool (MMAT) was used to evaluate the selected studies.

**Results:**

We identified four studies that presented findings of implementation outcomes research which were related to appropriateness, feasibility, and acceptability. Appropriateness was examined using knowledge scores (p = 0.022) and medication adherence scores (p < 0.05) that showed statistical significance between the control and intervention groups. Acceptability was assessed through a cross sectional quantitative descriptive study that evaluated the reasons for refusal and acceptance of treatment in a cohort of patients suffering from ESRD. Feasibility was examined in one cross sectional, and one mixed methods study that aimed to evaluate and understand the impact of initiating dialysis treatment and the feasibility of maintaining it in low-income families that care for children or adults with ESRD.

**Conclusion:**

The preliminary results of this review indicate a gap in the availability of implementation research studies about ESRD in Pakistan. The burden of ESRD, and the implementation methods by which it is treated is notable in Pakistan and requires evidence-based measures to be implemented to support the critical healthcare delivery platforms that provide treatment.

## 1. Introduction

Leveraging implementation research can help to strengthen healthcare structures and better define the barriers and facilitators of the programs or interventions that can provide the most effective outcomes. These facilitators can often include identifying models to improve healthcare service delivery or help streamline processes based on new evidence emerging in the field [1]. Chronic diseases, such as End Stage Renal Disease (ESRD) can often have individual and system-wide impacts on a country’s welfare especially when an individual’s quality of life is integrally connected to the access and cost of a frequent medical intervention such as dialysis. Low and middle income countries (LMIC) where ESRD is increasingly prevalent, like Pakistan, struggle with effective implementations across their clinical and non-clinical settings [2]. The development of chronic kidney disease (CKD) and its progression towards ESRD cause serious debilitation and reduced quality of life, resulting in significant premature death [2]. The nature of this type of morbidity in ESRD results in the need for a better understanding of how research is conducted, programs designed to address these problems and determine the best strategies to support those with long term illnesses that are dependent on critical healthcare delivery services. This paper seeks to better understand implementation research strategies around ESRD which can be used to improve system-wide approaches in many settings, particularly low-resource ones.

Patients diagnosed with ESRD typically have a long genesis of the illness and depend on healthcare delivery services once diagnosed. Some key characteristics that are precursors to the disease include the presence of kidney damage markers, proteinuria, and the glomerular filtration rate (GFR), along with abnormalities in kidney function define CKD, and ultimately ESRD [2]. CKD can be classified into five stages based on GFR level [2]. Treatment and management of ESRD is complex and requires attempts to correct the parameters causing the renal disfunction. Unfortunately, lifestyle modifications and management may not always be possible, especially in LMICs where access to healthcare is challenging for most patients. Long-term renal replacement therapy including haemodialysis or renal transplantation might be required in more advanced cases of renal disease [2].

In more heterogeneous societies like the United States (US) or Europe where the underlying cause of ESRD is often a chronic disease like diabetes mellitus, ESRD can be addressed through enhancing services and patient care management [2]. The adjusted prevalence of CKD stages 1 to 5 varied between 3.31% in Norway and 17.3% in northwest Germany and the adjusted prevalence of CKD stages 3 to 5 varied between 1.0% in central Italy and 5.9% in northwest Germany and seem to have a relationship with behaviors, lifestyle, and geographic variation [3]. Comparatively, there are a variety of underlying reasons for why ESRD progresses in more homogenous developing nations. In Pakistan, immune disease complications like chronic glomerulonephritis is the most common cause of ESRD [4]. Because the treatments for ESRD such as haemodialysis and renal transplant are costly and inaccessible for most of the population in rural areas, a significant percentage of the population in Pakistan die in the early parts of their treatment [5].

One review previously identified that the prevalence of CKD among participants (measured by the GFR) in four South Asian countries was the highest in Pakistan at 23.3% compared to just 10.6% in Nepal [6]. One study in the Peshawar region of Pakistan found that ESRD was 11.04 times more prevalent in diabetes patients, 7.29 less in non-hypertensive patients, and 3.1 times more likely in glomerulonephritis patients [7]. However, the overall extent of patients living with ESRD in Pakistan is still largely unknown, but studies have estimated an incidence rate of 152 per million [8]. Pakistan is in a unique position due to the lack of registries as well as the lack of timely death certificates with a clear cause of death coupled with patients who rarely seek medical attention contributes to an unknown burden of illness. In addition, the mean age of patients requiring renal replacement therapy in Pakistan is lower than that of higher income countries, impacting a younger population [8]. The current body of research around implementation research for the clinical management of patients with ESRD in Pakistan is limited and programs that have had success in regions like Pakistan are not widely available. This could be due in part to the lack of implementation science practice and principles in public health education requirements in Pakistan’s Health Services Academy [9].

The aim of this study is to conduct a systematic review analysis to identify and evaluate the available literature on implementation science outcomes research in relation to ESRD in Pakistan, which is burdened by these high rates of the condition. This systematic review seeks to understand the impact of implementation research strategies used among patients with ESRD in Pakistan, and identify studies that presented data on implementation science outcomes. Further, we compare and evaluate the implementation outcomes between the various studies.

## 2. Methods

### 2.1 Search strategy

A systematic database search of seven separate search engines was performed to detect all the relevant articles required for this review through October 22^nd^, 2022. The first chosen search engine was PubMed as this database includes articles related to biomedical research, clinical practice, and overall healthcare services. The second search engine used was the Web of Science which provides information from various high-quality multidisciplinary research journals. The third search engine was EMBASE which provides articles related to biomedical research as well as access to journals that cover numerous health related disciplines. The fourth search engine was Cochrane Library which is a collection of databases related to research in medicine and healthcare. The fifth search engine was the Cumulative Index to Nursing and Allied Health Literature (CINAHL) database that includes relevant journals from nursing and healthcare. The sixth search engine used was the Global Health engine by Ovid, a dedicated public health research database. The seventh and last search engine included all available datasets from Ovid, a complete and combined healthcare research platform.

Specific keywords were used to detect all the relevant articles available about ESRD in Pakistan. All seven search engines were searched for the relevant keywords in the titles and abstract sections. This search strategy ensures that we will have an adequate number of articles to screen for our review of implementation science outcomes research about ESRD in Pakistan. The list of the used keywords included terms such as “Pakistan, Islamic Republic of Pakistan, end stage kidney disease, ESRD, chronic kidney failure, chronic kidney disease, end stage renal failure, chronic renal disease, chronic renal insufficiency.” Furthermore, medical subject headings (Mesh) were used in search engines that supported such a function like PubMed and Cochrane Library. Lastly, truncations and Boolean terms ‘AND’ and ‘OR’ were used to expand and limit the searches when needed.

### 2.2 Inclusion and exclusion criteria

Articles were included if they: focused on implementation research conducted in or about Pakistan, ESRD, outcome evaluations and results related to acceptability, adoption, appropriateness, cost, feasibility, fidelity, penetration, and sustainability. Items included were implementation research studies conducted in or about Pakistan, implementation research studies conducted about ESRD, and implementation research studies that included implementation outcome evaluations and results such as, but not limited to, acceptability, adoption, appropriateness, cost, feasibility, fidelity, penetration, and sustainability. This review excluded implementation research studies not conducted in or about Pakistan, implementation research studies not conducted about ESRD, and research studies that did not include implementation outcomes. Systematic reviews or meta-analyses, abstracts, editorial papers, non-research-based articles, nonhuman studies, and mathematical modeling articles were also excluded. Based on this, the systematic review would yield papers and results that were very specific about implementation research in Pakistan around ESRD.

### 2.3 Data extraction and synthesis

The articles were screened by their titles and abstracts to identify articles that fit against our inclusion and exclusion criteria. Articles that did not provide sufficient information in the title or abstract sections were evaluated through a full text review. A screening and data extraction tool, Covidence, was used to import all the search results from the seven used search engines to find all the required articles. Each article was screened and extracted by two reviewers, with a third reviewer responsible for resolving conflicts when necessary.

### 2.4 Quality assessment

Articles included in this systematic review underwent critical appraisal of the literature using the 2018 version of the Mixed Methods Appraisal Tool (MMAT) [10]. The MMAT allows us to appraise various types of studies such as qualitative, quantitative randomized controlled trials, quantitative non-randomized trials, quantitative descriptive, and mixed methods studies. The appraisal process was achieved using three independent reviewers who evaluated the articles against the provided criteria in the MMAT.

To achieve this each reviewer had to screen each of the chosen articles against two screening questions that looked at whether the study had a clear research question, and if the collected data in that study allowed to address the studies research question. Once screening was completed, each study was evaluated using a provided MMAT algorithm that helped in selecting the correct criteria for the appraisal of each study. Lastly, each of the possible five categories had methodological quality assessment criteria explanations and examples that were used in the assessment process of each section of the selected criteria.

### 2.5 Project registration

This study was registered on the Open Science Framework (OSF) on the 31^st^ of January 2023 (https://doi.org/10.17605/OSF.IO/KTWG6).

## 3. Results

### 3.1 Search strategy results

**Fig 1:**
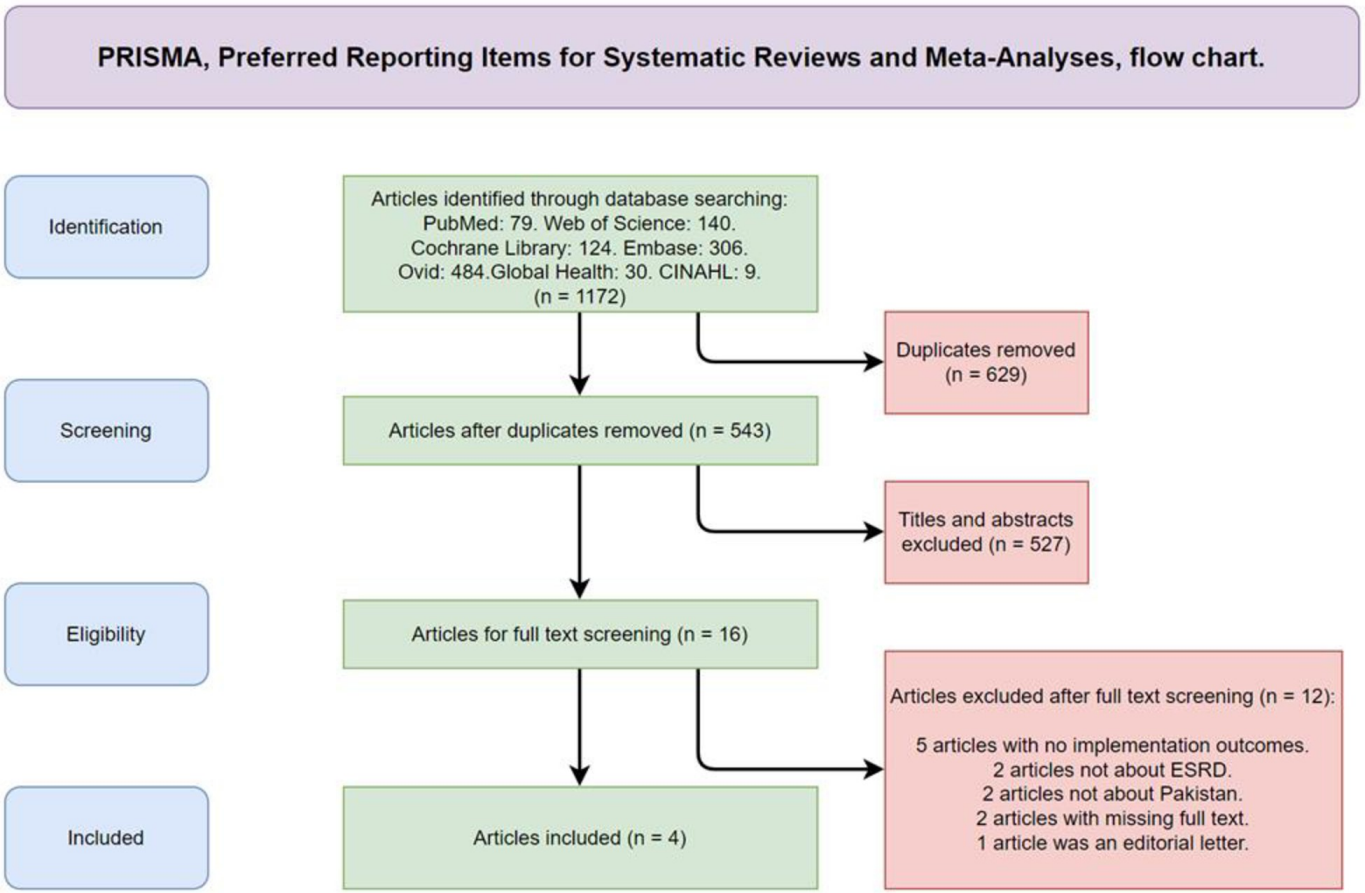
Preferred reporting items for systematic reviews and meta-analyses (PRISMA) flow chart.

The results of our search strategy based on each used search engine provided us with a total of 1172 articles to consider. These articles were screened through their titles, abstracts, and full text, and the detailed description of that process could be seen in Fig 1 which shows the Preferred Reporting Items for Systematic Reviews and Meta-Analyses (PRISMA) flow chart.

### 3.2 Results of the screening and data extraction process

The articles identified from the searched database engines were uploaded to a screening and data extraction tool named Covidence. This upload resulted in a total of 1172 articles of which 629 were duplicates, yielding 543 articles to screen for inclusion eligibility in the systematic review. Out of the 543 screened through their title and abstract against our inclusion and exclusion criteria, 527 studies were eliminated. This was achieved through voting on each article on Covidence by two reviewers with the third reviewer resolving all conflicts. Ultimately, 16 studies were assessed for eligibility through full-text evaluation and 12 were excluded due to meeting the requirements of our exclusion criteria (Fig 1). The remaining four articles that met our inclusion criteria are presented below.

### 3.3 Implementation research outcomes from the included studies

The evaluation of the full text articles revealed an overall lack of implementation research articles related to ESRD in Pakistan. Despite the lack of implementation science research articles, we identified a number of implementation outcomes listed in the table below (Table 1). The implementation outcomes were related to appropriateness in one article, feasibility in two articles, and acceptability in one article. Thus, these implementation outcomes were defined to establish their appropriateness for our systematic review. Acceptability was defined as the perception among involved patients that the intervention is agreeable, palatable, or satisfactory to them. Appropriateness was defined as the perceived fit of haemodialysis to address ESRD. Lastly, feasibility was defined as the extent to which the haemodialysis can be successfully used or conducted within the financially restrained settings in Pakistan [11].

**Table 1:** The implementational research outcomes of the included articles in this systematic review.

Author, Year	Location	Aim	Study Design	Study Period	Sample size	Measured Implementation Outcome	Appraisal Tool (MMAT)
Khokhar et al. (2020)	Lahore, Pakistan	To evaluate effectiveness of pharmacist intervention model in pre-dialysis CKD patients visiting nephrology outpatient department.	Multi-arm pre and post prospective non-randomized experimental study.	October 2018 to December 2018	120 participants	Appropriateness: Improvement in knowledge score upon follow up between intervention and control groups (19.10 ± 3.65 versus 17.57 ± 3.55, p = 0.022). Medication adherence score of the intervention group significantly improved as compared to the control group (p < 0.05).	High-moderate appraisal in the quantitative non-randomized criteria section (4/5).
Lanewala & Shekhani (2022)	Karachi, Pakistan	To understand the impact of dialysis treatment on the families with children suffering from ESRD.	Mixed Method—Cross Section Survey	2016	103 participants	Feasibility: 62% of the families have only one breadwinner. No other source in 63% Family contribution 19% Distance traveled 26 to 50 KM (65%) 70% use public transport Traveling up to 50 km, at a cost USD (5 ± 4). Spending 6 to 8 hours traveling to a dialysis session on an average.	High appraisal in the qualitative, descriptive quantitative, and mixed methods criteria sections (13/15).
Nafees et al. (2022)	Dialysis center of Allied Hospital Faisalabad, Pakistan.	Understand the impact of dialysis treatment on the family and individuals suffering from ESRD.	Quantitative, non-randomized cross-sectional study.	December 2021.	105 participants	Feasibility: Effect of ESRD on economic activities of patients was: - Not at all 4.76%. - To some Extent 6.67%. - To a greater extent at 88.57%. 86.67% of patients need assistance with dialysis 46.67% need to drive more than 50 KM 84.76% use public transport	High-moderate appraisal in the quantitative descriptive criteria section (4/5).
Shafi et. al (2018)	Tertiary Nephrology Hospital, Pakistan.	Understand the factors that influence acceptance and refusal of haemodialysis treatment in patients in Pakistan.	Quantitative, non-randomized cross-sectional study.	6 months	125 participants	Acceptability: The study showed that among hospitalized CKD patients who had indications to undergo HD - 57.6% of patients accepted, and 42.4% refused HD. 53.6% of all patients had used some form of alternative therapy Trust in doctor’s advice 86.1% was the most common reason for acceptance. Reasons for refusal varied from: - Financial 24.5%, fear of haemodialysis catheter 33.9%, and fear of AV fistula needles 24.5%. - No haemodialysis center near residence 11.3%	High-moderate appraisal in the quantitative descriptive criteria section (4/5).

The study by Khokhar et al [12] addressed the appropriateness outcome by evaluating the effectiveness of the pharmacist intervention model in pre-dialysis ESRD patients, and it was established in this study that the model improved patient’s health outcomes [12]. This study of 120 participants revealed that improvement in knowledge score upon follow-up between intervention and control groups was statistically significant (19.10 ± 3.65 versus 17.57 ± 3.55, p = 0.022) to lead to medication adherence score improvement when compared to the control group (p < 0.05) [12].

The study by Shafi et al [13] addressed the acceptability implementation outcome by evaluating the reasons for acceptance and refusal of haemodialysis treatment in patients. This cross-sectional study of 125 participants showed that among hospitalized patients with ESRD who were indicated for haemodialysis, 57.6% accepted the procedure, while 42.4% refused it. While trust in doctor’s advice (86.1%) was found to be the most common reason for acceptance, it is worth noting that 53.6% of all patients had used some form of alternative therapy due to the financial burden associated with this disease in 24.5%, or due to fear of the procedure like the haemodialysis catheter (33.9%) or the fear of the Arteriovenous fistula needles in 24.5%. Furthermore, geographical access to haemodialysis centers was found to be low in this study, with only 11.3% of the patients having such a center in their immediate residence area [13].

The studies by Lanewala & Shekhani [14] and Nafees et al [15] addressed the feasibility of implementation research outcomes in Pakistan. Both of these studies aimed to evaluate and understand the impact of initiating dialysis treatment and the feasibility of maintaining it in low-income families that care for children or adults with ESRD. The study by Lanewala & Shekhani [14] highlighted that the cost and expenditure on dialysis has been estimated to be between US$ (1680 and 2000) per year, which leads to added strain on families especially when 62% of the families have only one breadwinner that makes between 110 and 500 US$. This financial burden leads to further strains as up to 63% of patients and their families have no other source of income, and only about 19% depend on extended family contribution to support their treatment. Furthermore, 70% of participants in the study by Lanewala & Shekhani [14] used public transport to travel between 26 to 50 Kilometers (KM) for their dialysis treatment centers. Similar findings were also found in the article by Nafees et al [15] that looked into the ramifications of undergoing dialysis in adults from low-income settings. The authors reported that up to 88.57% of study participants indicated that ESRD affected their economic activities to a greater extent, and approximately 86.67% of the study participants expressed the need for assistance and help with the physical and financial responsibilities of undergoing dialysis. Additionally, the distance to the dialysis centers was deemed an added burden as 46.67% lived more than 50 KM from their treatment center and 84.76% of patients depended on public transport to reach those centers.

### 3.4 Quality of evidence and included studies limitations

The included studies highlight the lack of peer-reviewed implementation research articles specific to ESRD in Pakistan. While the study designs and outcomes measured some implementational outcomes, there was a dearth of rigorous design that addressed things beyond the observational aspects described above. All of the included articles were assessed for possible bias and confounding due to their study parameters. All the included studies (Table 1) lacked a representative sample size which could influence the generalizability of findings. A second limitation could be found in the duration of the studies which varied from an undefined period to 6 months, and this time limitation could have introduced bias. A third source of limitation could be related to selection bias and interviewer bias as not all studies defined how they addressed these possible confounders.

While the included studies could be suspected of bias, it should be noted that the critical appraisal of these articles using the MMAT showed an overall high to high-moderate appraisal scores in through the evaluated criteria. The first included study by Khokhar et al [12], showed high-moderate appraisal in the quantitative non-randomized criteria section with four out five parameters clearly explored in the article, but still missing a clear clarification of how they addressed confounders in the study. The second included study by Lanewala and Shekhani [14], showed high appraisal in the qualitative, descriptive quantitative, and mixed methods criteria sections with 13 out of 15 parameters clearly explored in the article, but still missing a clear clarification of how they dealt with divergences as well as clearly not having a representative sample. The third and fourth included articles by Nafees et al [15] and Shafi et al [13], both showed high-moderate appraisal in the quantitative descriptive criteria section with four out of five parameters clearly explored in those articles, but still missing a clear clarification if the measurements were appropriate in both those studies [12–15].

## 4. Discussion

This review sought to understand the impact of implementation research strategies on ESRD among patients living in Pakistan. Final articles included in this review identified acceptability, feasibility, and appropriateness as the most common opportunities for intervention related to ESRD in Pakistan. The four papers, which are undoubtedly useful and important reviews of their clinical and healthcare related efficacy, provide potential directions for implementation research. It is evident that more research is needed in the utilization of implementation research strategies on ESRD among patients living in Pakistan, with only four studies identified in our systematic review across various health disciplines. Furthermore, our limited results indicate that the prominent research design for ESRD patients in Pakistan was cross sectional with 75% (n = 3) of the studies included utilizing a cross sectional design in their methodology. Additionally, of the studies reviewed, the majority described the significant financial burden on families which affects the feasibility of sustaining haemodialysis as a treatment option for those who are disadvantaged, marginalized, and living in poverty.

This systematic review indicates that the body of research around ESRD in Pakistan, specifically related to implementation science outcomes is limited. Implementation research teams have worked to engage Pakistani government officials in the benefits of implementation research and applications, particularly for health policy and healthcare delivery. Shahabuddin et al [16] noted that the partnership between the Government of Pakistan, United Nations International Children’s Emergency Fund (UNICEF), and the Alliance for Health Policy and Systems Research partnered together in 2016 to launch the country’s first embedded implementation research initiative for their immunization programs. Their findings suggested that there was significant value added for health officials in Pakistan to ensure the successful implementation of key health programs and that other implementation research initiatives could benefit from clarifying roles and responsibilities of various stakeholders [16]. The result of this systematic review further underscores the need for more thorough implementation research studies around ESRD in Pakistan.

There are several limitations to this review. Due to the small sample (n = 4), it is difficult to identify clear trends and themes that would help to understand the full impact of implementation research in Pakistan for ESRD patients. However, with the limited studies included, half of them (n = 2) discussed implementation outcomes related to feasibility. Furthermore, this review suggests that future implementation strategies around ESRD within patient populations in Pakistan are needed to support the programs that help the country’s most vulnerable population receive critical services like haemodialysis.

## 5. Conclusion

The review is one of the first systematic reviews of implementation research outcomes for patients living with ESRD in Pakistan. The approach taken by this study yielded four studies that included mixed method, quantitative descriptive, and quantitative nonrandomized styles. The preliminary results of this review indicate a gap in the availability of implementation research studies about ESRD in Pakistan. The burden of ESRD, and the implementation methods by which it is treated is notable in Pakistan and requires evidence-based measures to be implemented to support the critical healthcare delivery services that provide those treatment services. To reduce this burden, there is an opportunity to partner with key stakeholders to increase awareness of implementation research to inform health policies with the Pakistani government and their healthcare system.

## Supporting information

S1 Checklist (DOCX)
